# Association of HbA1c with hospitalization and mortality among patients with heart failure and diabetes

**DOI:** 10.1186/s12872-016-0275-6

**Published:** 2016-05-20

**Authors:** Saul Blecker, Hannah Park, Stuart D. Katz

**Affiliations:** Department of Population Health, NYU School of Medicine, New York, NY USA; Department of Medicine, NYU School of Medicine, New York, NY USA

**Keywords:** Heart failure, Diabetes, hbA1c

## Abstract

**Background:**

Comorbid diabetes is common in heart failure and associated with increased hospitalization and mortality. Nonetheless, the association between glycemic control and outcomes among patients with heart failure and diabetes remains poorly characterized, particularly among low income and minority patients.

**Methods:**

We performed a retrospective cohort study of outpatients with heart failure and diabetes in the New York City Health and Hospitals Corporation, the largest municipal health care system in the United States. Cox proportional hazard models were used to measure the association between HbA1c levels and outcomes of all-cause hospitalization, heart failure hospitalization, and mortality.

**Results:**

Of 4723 patients with heart failure and diabetes, 42.6 % were black, 30.5 % were Hispanic/Latino, 31.4 % were Medicaid beneficiaries and 22.9 % were uninsured. As compared to patients with an HbA1c of 8.0–8.9 %, patients with an HbA1c of <6.5, 6.5–6.9, 7.0–7.9, and ≥9.0 % had an adjusted hazard ratio (aHR) (95 % CI) for all-cause hospitalization of 1.03 (0.90–1.17), 1.05 (0.91–1.22), 1.03 (0.90–1.17), and 1.13 (1.00–1.28), respectively. An HbA1c ≥ 9.0 % was also associated with an increased risk of heart failure hospitalization (aHR 1.33; 95 % CI 1.11–1.59) and a non-significant increased risk in mortality (aHR 1.20; 95 % CI 0.99–1.45) when compared to HbA1c of 8.0–8.9 %.

**Conclusions:**

Among a cohort of primarily minority and low income patients with heart failure and diabetes, an increased risk of hospitalization was observed only for an HbA1c greater than 9 %.

## Background

Heart failure and diabetes are two chronic diseases that commonly coexist. Among patients with heart failure, the prevalence of diabetes is as high as 40 % [1–3] and is increasing [4]. The high rate of diabetes in heart failure holds clinical significance as diabetes has been associated with worse outcomes in heart failure. For instance, in a meta-analysis of 17 studies, patients with heart failure and diabetes were found to have a 28 % increase in mortality and a 36 % increase in hospitalizations as compared to patients with heart failure but no diabetes [5]. The relationship between heart failure and diabetes may be magnified among high-risk populations such as racial minorities, individuals of low socioeconomic status, and the uninsured [6–10].

Despite the increased risk of poor outcomes among patients with heart failure and diabetes, the optimal hemoglobin A1c (HbA1c) level for patients with heart failure and diabetes remains uncertain. Due to lack of existing data, heart failure guidelines do not specifically address the intensity of glycemic control among individuals with heart failure [11], and the American Diabetes Association (ADA) guidelines do not provide a specific treatment target [12]. Although elevated levels of HbA1c have been associated with increased risk for heart failure related hospitalizations [13–16], intervention trials in patients with Type 2 diabetes have failed to show benefit with tight glycemic control and some studies have even suggested harm [17–19]. Additionally, tight glycemic control could predispose to volume retention in heart failure by decreasing the osmotic diuretic effect of glucosemia [20]. Given the potential side effects of both tight and loose glycemic control among heart failure patients, moderate glycemic control would seem appropriate. Indeed, a study of primarily male patients from Veterans Affairs (VA) medical centers demonstrated a U-shaped relationship between HbA1c and mortality, with the lowest risk observed for patients with an HbA1c between 7.1 and 7.8 % [21]. Conversely, two studies from a single academic advanced heart failure clinic found low [22, 23] HbA1c values to be associated with increased risk of mortality, while a high HbA1c was associated with mortality for heart failure patients enrolled in the CHARM study [24]. Notably, these prior studies focused on select patient populations such as those who were fully insured or who agreed to participate in a clinical trial [21–24]. As a result, the optimal HbA1c target for heart failure patients with diabetes remains uncertain, especially for members of racial and ethnic minorities not well represented in previous studies [21–24].

The purpose of this study was to evaluate the association of HbA1c with all-cause hospitalization among patients with heart failure and diabetes in a hospital system serving a diverse, urban, and primarily low-income population. We hypothesized that hospitalization rates would vary with level of HbA1c with a U-shaped relationship, such that moderate glycemic control would be associated with reduced all-cause hospitalization, heart failure hospitalization, and mortality.

## Methods

We performed a non-concurrent cohort study of patients in the New York City Health and Hospitals Corporation (HHC), the largest municipal health care system in the United States. The corporation serves approximately 1.4 million people, of whom nearly 500,000 are uninsured, and includes 11 hospitals, six diagnostic and treatment centers, four long term care centers, and one hundred community health centers [25]. The primary data source for the study was the HHC data warehouse which contains data from the electronic health records (EHRs) from each of the hospitals and includes demographic information, clinical measures, problem lists, laboratory tests and results, and medication orders. Patient data from HHC was matched to two New York State data registries to obtain outcomes data: the New York State Vital Statistics and the New York Statewide Planning and Research Cooperative System (SPARCS). Vital statistics maintains records of deaths in the state, while SPARCS contains all acute care admissions for non-federal hospitals in the state.

We included patients with heart failure and diabetes who had an outpatient clinic visit in HHC between 1/1/2007 and 12/31/2010. Diagnoses were based on International Classification of Diseases, Clinical Modification (ICD-9-CM) codes of 428 and 250, respectively, in the problem list [3, 26]. Additional inclusion criteria were age 18 years and older and measurement of an HbA1c either on or up to 90 days prior to the clinic visit. For patients with multiple encounters, we only included the first visit for which inclusion criteria were met.

The primary outcome was all-cause hospitalization. Secondary outcomes were heart failure hospitalization and mortality. Heart failure hospitalization was based on a principal diagnosis of heart failure using standard ICD-9-CM codes [3, 27].

The primary exposure was HbA1c, which was categorized into the following clinically relevant categories: <6.5, 6.5–6.9, 7.0–7.9, 8.0–8.9, and ≥9.0 %. Other patient characteristics included age, gender, race, ethnicity, primary payer, prior utilization, comorbidities, diabetes severity, systolic blood pressure, pulse, creatinine, hemoglobin, active medications, prior utilization, and HHC facility of the clinic visit. Characteristics were assessed at time of clinic visit with the exception of laboratory values which, if unavailable at time of clinical visit, could be included if assessed up to 90 days prior to the clinic visit. In the data warehouse, race was self-reported as a single category and included responses of “Black or African American”, “Hispanic Black”, and “Hispanic or Latino”. From this information, we created a race variable that was categorized as black or other and an ethnicity variable which was categorized as Hispanic/Latino and other. Comorbidities were based on standard algorithms [28]. Diabetes severity was assessed using the diabetes complication severity index, a validated index of complications of diabetes that has been associated with hospitalization and mortality [29]. Medication utilization was based on an active prescription and included the following medication classes: loop diuretic, angiotensin converting enzyme (ACE) inhibitor or angiotensin receptor blocker (ARB), beta blocker, insulin, metformin, and sulfonylurea. Prior utilization included hospitalization in the prior 90 days and number of hospitalizations, emergency department visits, and clinic visits in the prior year.

Follow up time was defined from time of clinic visit. Patients were followed to the time of death or December 31, 2010; patients who were unable to be accurately matched to mortality data (*n* = 117) were censored on their last date of service.

### Statistical analysis

Differences in frequency of outcomes among all HbA1c groups were compared using Pearson chi-squared tests. We plotted Kaplan-Meier curves for hospitalization; curves were compared using the log-rank test. Cox proportional hazard models were developed to determine the relative association of HbA1c category with the primary outcome variable, all-cause hospitalization. The proportionality assumption was evaluated graphically using the -log(−log(survival function)) plot and statistically using the Schoenfeld residuals. We developed adjusted Cox models incorporating baseline patient characteristics as well as shared-frailty to account for clustering within each HHC site [30]. Covariates included in the adjusted model were age, sex, race, ethnicity, insurance, comorbidities, blood pressure, heart rate, creatinine, hemoglobin, medications, and prior utilization. Similar models were developed for the secondary outcomes. We performed a sensitivity analysis in which we developed similar models with the primary independent variable of HbA1c categorized as <6.5, 6.5–6.9, 7.0–7.9, 8.0–8.9, 9.0–9.9, 10.0–11.9, and ≥12.0 %.

Statistical significance was pre-specified with an alpha level of 0.05 (two-tailed). Statistical analyses were performed using Stata 13 (StataCorp, College Station, TX).

This study was approved by the New York University School of Medicine Institutional Review Board.

## Results

Of the 4723 patients with heart failure and diabetes included in the study, the median age was 64 years, 42.6 % were black, and 30.5 % were Hispanic or Latino. At the time of clinic visit, 31.4 % of patients were Medicaid beneficiaries and 22.9 % of patients had no insurance.

The mean HbA1c (SD) for the cohort was 8.2 (2.4). The distribution of HbA1c categories of <6.5, 6.5–6.9, 7.0–7.9, 8.0–8.9, and ≥9.0 % was: 21.8, 12.7, 22.6, 15.0, and 28.0 %, respectively. Patients with high HbA1c were younger and had high rates of Medicaid insurance when compared to patients with low HbA1c (Table 1). Patients in the lowest HbA1c group were most likely to be black and had the highest prevalence of kidney disease. Patients with higher HbA1c had higher rates of insulin use, while metformin was used most frequently among patients with HbA1c of 6.5–6.9 %. HbA1c appeared to be inversely correlated with number of outpatient encounters, as patients in the lowest two HbA1c groups had the highest average number of clinic visits. (Table 1).

**Table 1:** Baseline characteristics of 4723 patients with heart failure and diabetes, by hemoglobin A1c (HbA1c) category

	HbA1c range				
	<6.5	6.5–6.9	7–7.9	8–8.9	≥9
	(*n* = 1028)	(*n* = 600)	(*n* = 1065)	(*n* = 706)	(*n* = 1324)
Age, years	67.1 (12.9)	66.3 (12.4)	65.4 (12.0)	63.7 (11.7)	60.9 (11.5)
Female	53.6	52.0	55.6	52.3	49.2
Black Race	47.2	41.7	41.2	39.7	42.0
Hispanic/Latino Ethnicity	28.7	28.8	29.8	32.2	32.2
Insurance					
Medicaid	28.2	31.2	30.6	33.3	33.5
Medicare	30.5	27.0	25.7	24.1	18.7
Private	4.1	4.5	3.4	4.7	4.6
Self-Pay	18.0	18.8	22.4	24.9	27.7
Other	19.3	18.5	17.9	13.0	15.4
Diabetes Severity Index	3.5 (1.7)	3.3 (1.7)	3.3 (1.6)	3.5 (1.7)	3.4 (1.7)
Comorbid Conditions					
Myocardial Infarction	10.8	13.0	11.8	11.2	12.2
Peripheral Vascular Disease	14.7	11.7	13.3	15.0	11.7
Cerebrovascular Disease	17.8	15.5	16.9	13.2	10.7
Dementia	2.2	2.3	2.0	1.4	1.1
Chronic Pulmonary Disease	23.8	26.3	27.2	24.5	25.8
Rheumatic Disease	2.4	1.8	2.0	1.1	1.4
Liver Disease	7.5	5.2	4.0	3.7	4.8
Renal Disease	30.3	19.7	18.6	21.1	21.4
Malignancy or Tumor	8.6	10.0	6.4	6.5	5.8
Heart Rate, beats/min	74.8 (13.1)	75.2 (12.9)	76.3 (13.2)	77.6 (14.1)	78.2 (13.8)
Systolic Blood Pressure, mmHG	133.7 (22.4)	135.3 (23.0)	134.6 (22.2)	135.6 (23.4)	134.7 (22.9)
Creatinine, mg/dl	1.9 (1.8)	1.5 (1.2)	1.5 (1.2)	1.5 (1.3)	1.5 (1.3)
Hemoglobin, g/dl	11.6 (2.0)	12.0 (1.9)	12.0 (1.9)	12.1 (1.9)	12.2 (2.0)
ACE inhibitor or ARB	55.1	58.2	58.8	56.7	59.9
Beta-Blocker	36.7	34.8	37.5	35.6	34.5
Loop Diuretic	57.9	63.0	62.4	62.3	57.4
Insulin	21.7	25.2	35.8	50.9	62.3
Metformin	14.1	22.2	16.4	18.8	19.5
Sulfonylurea	9.0	11.7	12.4	9.8	10.6
Prior Utilization					
Hospitalization in Prior 90 Days	54.3	45.3	52.8	53.8	58.9
Hospitalizations, Prior Year	1.5 (2.0)	1.2 (1.8)	1.4 (1.9)	1.4 (1.7)	1.5 (2.0)
ED Visits, Prior Year	0.6 (1.3)	0.5 (1.1)	0.6 (1.7)	0.5 (1.3)	0.6 (1.3)
Clinic Visits, Prior Year	6.9 (12.8)	7.3 (15.3)	6.6 (15.0)	5.3 (12.6)	5.5 (12.2)

Values are percentages or mean (standard deviation)

**Fig. 1:**
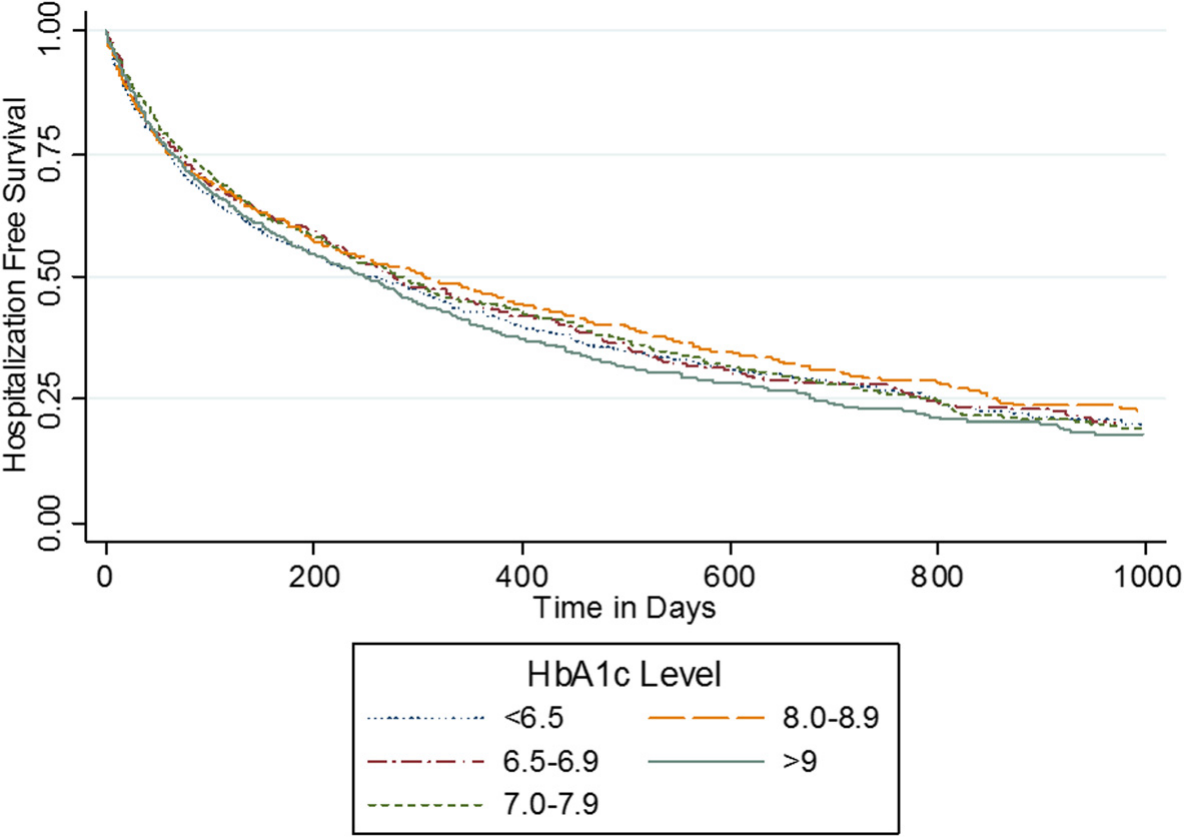
Kaplan Meier curves for hospitalization free survival by HbA1c category among 4723 patients with heart failure and diabetes. *p* = 0.10 for differences among curves using the log-rank test

For the primary outcome of all-cause hospitalization, patients were followed for a median (25^th^,75^th^ percentile) of 173 (46,452) days. During follow up, 67.0 % of patients were hospitalized for any cause. Overall rates of hospitalization were similar across HbA1c groups (Table 2). Similarly, we found no difference in the Kaplan Meier curves between HbA1c groups (see Fig. 1). In unadjusted analysis, an HbA1c ≥9 % was associated with increased risk of hospitalization, while an HbA1c < 6.5 % was associated with risk of hospitalization that did not reach statistical significance (Table 3). After adjusting for covariates, the lowest four categories of HbA1c (<6.5, 6.5–6.9, 7.0–7.9, and 8.0–8.9 %) were associated with similar hazard for hospitalization (Table 3). Only patients with an HbA1c ≥ 9.0 % had a significant increased risk for hospitalization (adjusted HR 1.13; 95 % CI 1.00–1.28) when compared to patients with HbA1c 8.0–8.9 % (Table 3).

**Table 2:** Frequency of outcome events of hospitalization, mortality, and heart failure hospitalization during follow up, by HbA1c category

	HbA1c range					*p*
	<6.5 (*n* = 1028)	6.5–6.9 (*n* = 600)	7–7.9 (*n* = 1065)	8–8.9 (*n* = 706)	≥9 (*n* = 1324)	
Hospitalization	68.6	64.7	65.2	66.2	68.9	0.16
Heart Failure Hospitalization	26.6	27.5	28.9	28.8	35.3	<0.001
Mortality	30.5	25.5	24.1	23.7	26.8	<0.01

**Table 3:** Hazard ratio for outcomes of hospitalization, mortality, and heart failure hospitalization by HbA1c category among 4723 patients with heart failure and diabetes. Adjusted for age, sex, race, ethnicity, insurance, comorbidities, blood pressure, heart rate, creatinine, hemoglobin, medications, and prior utilization

	Hospitalization		Heart failure hospitalization		Mortality	
	Unadjusted	Adjusted	Unadjusted	Adjusted	Unadjusted	Adjusted
HbA1c category						
<6.5	1.12 (0.99–1.26)	1.03 (0.90–1.17)	0.95 (0.79–1.14)	0.92 (0.75–1.13)	1.32 (1.10–1.60)	1.16 (0.94–1.42)
6.5–6.9	1.06 (0.93–1.22)	1.05 (0.91–1.22)	0.97 (0.79–1.20)	1.03 (0.83–1.29)	1.09 (0.87–1.35)	1.11 (0.88–1.41)
7–7.9	1.04 (0.93–1.18)	1.03 (0.90–1.17)	1.07 (0.89–1.28)	1.12 (0.93–1.36)	1.03 (0.85–1.25)	1.08 (0.88–1.33)
8–8.9	1 [ref]	1 [ref]	1 [ref]	1 [ref]	1 [ref]	1 [ref]
≥9	1.15 (1.03–1.29)	1.13 (1.00–1.28)	1.31 (1.11–1.55)	1.33 (1.11–1.59)	1.13 (0.94–1.36)	1.20 (0.99–1.45)

The rate of heart failure hospitalizations was 35.3 % for patients with HbA1c ≥ 9.0 % and 26.6–28.9 % for patients in categories with lower HbA1c (Table 2). After adjustment for covariates, HbA1c ≥ 9.0 % was associated with a hazard ratio for heart failure hospitalization of 1.33 (95 % CI 1.11–1.59) when compared to HbA1c 8.0–8.9 %. Other categories of HbA1c did not significantly differ in hazard ratio for heart failure hospitalization when compared to the reference (Table 3).

The median (25^th^,75^th^ percentile) follow up for mortality was 658 (289,1114) days. Over 30 % of patients with HbA1c < 6.5 % died during the period while mortality rates for higher HbA1c categories ranged from 23.7 to 26.8 % (Table 2). In unadjusted analysis, HbA1c < 6.5 % was associated with a 32 % (95 % CI 10–60 %) increase in hazard of death as compared to the reference group of HbA1c 8.0–8.9 %. However, this association was attenuated and was no longer significant after adjusting for covariates (adjusted HR 1.16; 95 % CI 0.94–1.42; Table 3). Similarly, an elevated HbA1c ≥ 9 % was associated with increased mortality in multivariable analysis that did not reach statistical significance (adjusted HR 1.20; 95 % CI 0.99–1.45; Table 3). Indeed, in multivariate adjustment, no level of HbA1c was statistically associated with increased mortality.

In sensitivity analysis in which we categorized higher HbA1c values into 9.0–9.9 %, 10.0–11.9 %, and ≥12 %, we found that hospitalization risk was similar for each of these individual categories as when grouped together (Appendix). An HbA1c over 12 % was associated with a significant increase in mortality as compared to HbA1c 8.0–8.9 %, with an adjusted HR 1.33 (95 % CI 1.03–1.73). We found no significant association between HbA1c of 9.0–9.9 % or 10.0–11.9 % and mortality (Appendix).

## Discussion

In this cohort of patients with heart failure and diabetes from a diverse, urban, and primarily low-income hospital system, we found no difference in risk of hospitalization or mortality for HbA1c levels up to 8.9 %. Hospitalization risk only increased at the highest HbA1c level; an HbA1c of 9.0 % or higher was associated with a 13 % increase in the relative hazard of all-cause hospitalization and a 33 % increase in the relative hazard of heart failure hospitalization when compared to lower HbA1c values. Furthermore, we found the association between HbA1c and mortality to be significant only for HbA1c levels above 12 %. These findings suggest that, in this patient population, glycemic control is not a strong predictor of outcomes in heart failure and diabetes and risk of hospitalization begins to increase only when HbA1c ≥ 9 %.

Consistent with our initial hypothesis, that both high and low levels of HbA1c would be associated poor outcomes, we found that an HbA1c < 6.5 % was associated with increased risk of mortality and a nonsignificant increase in hospitalization in unadjusted analysis. However, a low HbA1c was also associated with a number of factors associated with poor outcomes, including increased age, cerebrovascular disease, and creatinine. After adjusting for such confounders, a low HbA1c was no longer associated with mortality or hospitalization. Some studies have found that HbA1c may have a U- or J-shaped relationship to outcomes [21, 31, 32], suggesting there may also be a low threshold, below which risk increases [32]. In this population of patients with heart failure and diabetes, any risk associated with low HbA1c was explained by other factors. These findings do not support a low threshold effect in patients with heart failure and diabetes.

In our study, only levels of HbA1c of 9 % or higher were associated with hospitalization and mortality. Conversely, observational studies in the general population have suggested that mortality risk increases when the HbA1c rises above 7 % [33, 34], the level recommended by the ADA for most patients with diabetes [12]. Results of four prior studies of the association between HbA1c and health outcomes among patients with heart failure and diabetes have been mixed. Two studies of patients with advanced heart failure from the same single center suggested that tighter glycemic control was associated with increased mortality [22, 23]. Conversely, among a subgroup of patients with heart failure and diabetes enrolled in the CHARM study, Gerstein and colleagues found that a 1 % increase in HbA1c was associated with an increased hazard ratio of 1.13 for heart failure hospitalization and 1.11 for mortality [24]. Finally, Aguilar and colleagues studied 5815 male patients in the VA and found that an HbA1c of 7.1–7.8 % was associated with a hazard ratio for mortality of 1.31–1.45 as compared to both higher and lower values. However, the authors found no significant association between HbA1c and hospitalization [21].

While the studies by Gerstein [24] and Aguilar [21] found higher HbA1c was associated with increased risk of mortality, we found no statistical association between HbA1c and mortality in our primary analysis. The difference between our study and the prior studies is that we included a real-world patient population with an even gender mix and high rates of patients who were uninsured and of racial/ethnic minorities; studies by Gerstein and Aguilar included more selective subjects who were enrolled in a clinical trial or were primarily male and insured through the VA, respectively. Nonetheless, we do not believe findings in our study can be fully explained by racial or ethnic mix. Although both black and Hispanic patients are known to have higher levels of HbA1c as compared to white patients [35–37], HbA1c has been shown to have a similar relationship for clinical outcomes among different racial groups [36, 38] and the same target HbA1c has been recommended for all patients with diabetes, regardless of race [12].

Our findings should be interpreted in the context of study limitations. First, inclusion was based on diagnostic coding algorithms and may be subject to misclassification despite using validated diagnostic codes [39, 40]. Second, although the HHC database contains extensive data on vital signs, inpatient and outpatient claims, medication utilization and laboratory data, it may lack data on several potentially important variables, including ejection fraction and functional status. As a result, we were unable to determine of HbA1c had a differential association with outcomes for patients with heart failure with reduced versus preserved ejection fraction or patients with type 1 versus type 2 diabetes. Third, as we were relied on real world EHR data, some of our data may be subject to misclassification, such as self-reported race field. Fourth, the data relied on the EHR within a single hospital system so we may have missed medication prescriptions and clinical encounters that occurred at another facility. Fifth, as we relied on a state registry for hospitalization information, we may have missed some hospitalizations that occurred out of state. Sixth, the median follow up for mortality was 658 days. Nonetheless, as the mortality rate was over 25 % in this study, the null finding of association between HbA1c and mortality was probably not fully attributable to low event rate.

Our study had a number of unique strengths. We included patients from a large public health system which serves many uninsured and minority patients in real world practice settings. Such patients are frequently underrepresented in clinical trials [41]. Additionally, we linked our EHR data to state registry data to obtain complete hospitalization follow up. This is notable as a large number heart failure related hospitalizations are hospitals other than where care is primarily obtained [42].

## Conclusions

We found the associated risk of hospitalization increases for HbA1c ≥ 9 %, and not lower HbA1c values, among patients with heart failure and diabetes. The ADA currently recommends targeting an HbA1c < 7 % for most patients with diabetes but suggests that “less stringent A1c goals (such as < 8 %) may be appropriate” for subpopulations such as those with limited life expectancy or significant comorbidity [12]. In support of less stringent glycemic target for patients with heart failure and diabetes, our data suggest that there may be a weak threshold effect of HbA1c above 9 % in this population. Nonetheless, randomized control trials are needed to determine the recommended HbA1c for patients with heart failure and diabetes, who are at high risk of hospitalization and mortality.

## Ethics approval and consent to participate

This study was approved by the NYU School of Medicine institutional review board, which granted a waiver of consent.

## Consent for publication

Not applicable.

## Availability of data and materials

Data will not be shared due to restrictions related to patient confidentiality.

## Appendix

**Table 4:** Hazard ratio for outcomes using further stratification of HbA1c vlues. Adjusted for age, sex, race, ethnicity, insurance, comorbidities, blood pressure, heart rate, creatinine, hemoglobin, medications, and prior utilization

	Hospitalization		Heart failure hospitalization		Mortality	
	Unadjusted	Adjusted	Unadjusted	Adjusted	Unadjusted	Adjusted
HbA1c Category						
<6.5	1.12 (0.99–1.26)	1.03 (0.90–1.17)	0.95 (0.79–1.14)	0.92 (0.75–1.13)	1.32 (1.10–1.60)	1.16 (0.94–1.42)
6.5–6.9	1.06 (0.93–1.22)	1.05 (0.91–1.22)	0.97 (0.79–1.20)	1.03 (0.83–1.29)	1.09 (0.87–1.35)	1.11 (0.88–1.41)
7–7.9	1.04 (0.93–1.18)	1.03 (0.90–1.17)	1.07 (0.89–1.28)	1.12 (0.93–1.36)	1.03 (0.85–1.25)	1.08 (0.88–1.33)
8–8.9	1 [ref]	1 [ref]	1 [ref]	1 [ref]	1 [ref]	1 [ref]
9–9.9	1.14 (0.99–1.32)	1.10 (0.94–1.28)	1.23 (0.99–1.52)	1.22 (0.98–1.53)	1.14 (0.90–1.44)	1.15 (0.90–1.48)
10–11.9	1.15 (1.00–1.33)	1.17 (1.01–1.36)	1.27 (1.03–1.57)	1.39 (1.12–1.73)	1.07 (0.85–1.34)	1.16 (0.91–1.48)
≥12	1.16 (0.99–1.36)	1.11 (0.94–1.31)	1.47 (1.18–1.82)	1.38 (1.09–1.74)	1.22 (0.95–1.56)	1.33 (1.03–1.73)

## Abbreviations

HbA1c: hemoglobin A1c
ADA: American Diabetes Association
VA: Veterans Affairs
HHC: New York City Health and Hospitals Corporation
ICD-9-CM: International Classification of Diseases, Clinical Modification
ACE: angiotensin converting enzyme
ARB: angiotensin receptor blocker
HR: hazard ratio
CI: confidence interval
EHR: electronic health record
